# Genetic Association Study of *TNFAIP3*, *IFIH1*, *IRF5* Polymorphisms with Polymyositis/Dermatomyositis in Chinese Han Population

**DOI:** 10.1371/journal.pone.0110044

**Published:** 2014-10-22

**Authors:** Si Chen, Qian Wang, Ziyan Wu, Yuan Li, Ping Li, Fei Sun, Wenjie Zheng, Qingjun Wu, Chanyuan Wu, Chuiwen Deng, Fengchun Zhang, Yongzhe Li

**Affiliations:** Department of Rheumatology and Clinical Immunology, Peking Union Medical College Hospital, Chinese Academy of Medical Sciences & Peking Union Medical College, Key Laboratory of Rheumatology and Clinical Immunology, Ministry of Education, Beijing, China; The University of Hong Kong, China

## Abstract

**Background:**

Single-nucleotide polymorphisms (SNPs) in the *TNFAIP3*, *IFIH1*, and *IRF5* genes have been associated with several auto-inflammation diseases, while the susceptibility between these genes and idiopathic inflammatory myopathies (IIMs) were not reported. This study aimed to investigate whether *TNFAIP3*, *IFIH1*, and *IRF5* gene polymorphisms confer susceptibility for the IIMs in Chinese Han population.

**Methods:**

A large case–control study of Chinese subjects with polymyositis (PM) (n = 298) and dermatomyositis (DM) (n = 530) was accomplished. 968 healthy and ethnically matched controls were available for comparison. Six SNPs in the *TNFAIP3* region (rs2230926 and rs5029939), the *IFIH1* gene (rs1990760 and rs3747517) and the *IRF5* region (rs4728142 and rs729302) were assessed and genotyped using the Sequenom MassArray iPLEX platform.

**Results:**

Our study indicated a strong allele association was observed in PM/DM and PM patients for rs2230926 (OR: 1.61, 95%CI: 1.20–2.16, *P_c_* = 7.5×10^−3^; OR: 1.88, 95%CI: 1.30–2.74, *P_c_* = 4.0×10^−3^, respectively) and rs5029939 (OR: 1.64, 95%CI: 1.21–2.21, *P_c_* = 6.0×10^−3^; OR: 1.88, 95%CI: 1.28–2.76, *P_c_* = 5.5×10^−3^,respectively). And rs2230926 and rs5029939 were significantly associated with interstitial lung disease (ILD) in PM/DM and PM patients (*P_c_* = 0.04 and *P_c_* = 0.016; *P_c_* = 0.02 and *P_c_* = 0.03, respectively). In addition, rs4728142 allele and genotype had significant association with PM/DM patients (*P_c_* = 0.026 and *P_c_* = 0.048, respectively). Further analysis with three logistic regression genetic models revealed statistically significant difference in the genotypic distribution in the PM/DM, PM or DM patients when the additive and dominant models were used.

**Conclusions:**

This was the first study to reveal *TNFAIP3* and *IRF5* polymorphisms were associated with PM/DM patients or these patients with ILD, indicating that *TNFAIP3* and *IRF5* might be the susceptibility gene for PM/DM patients in Chinese Han population.

## Introduction

The idiopathic inflammatory myopathies (IIMs) are a heterogeneous group of rare systemic disease characterized by skeletal muscle weakness and presented with extra-muscular manifestations such as skin rashes, interstitial lung disease (ILD) and malignancy [1]. The primary subgroups of IIMs are polymyositis (PM), dermatomyositis (DM) and inclusion-body myositis (IBM). Although IIMs are generally considered as multi-factorial autoimmune disease, the etiology of IIMs remains mostly unclear. Myositis patients may develop additional rheumatic diseases, and the occurrence of autoimmune disorders in near relatives is higher [2]–[3]. According to the understanding of other autoimmune diseases, it is supposed that IIMs development may be a result of both genetic and environmental factors or their interactions. Therefore, it is possible to make use of the extensive knowledge of other rheumatic diseases that share pathogenic traits with IIMs to obtain insight into the probable genetic intricacy of IIMs.

To date, the abundant evidence demonstrated that substantial genetic risk for IIMs existed within the major histocompatibility complex (*MHC*) gene region [4], whereas only a handful of non-MHC loci were identified by genome-wide association study (GWAS) and candidate gene association studies in Japanese and European populations [5]–[15]. Tumor necrosis factor alpha (*TNF-α*) [5]–[8], interleukin (*IL*)*-1α*, *IL-1β* [7], interferon (*IFN*)*-γ* [9], interferon-induced helicase (*IFIH1*) [10], mannose-binding lectin 2 (*MBL2*) [11], protein tyrosine phosphatase N22 (*PTPN22*) [12], and signal transducer and activator of transcription 4 (*STAT4*) [13], and other genes were indicated to associate with the risk of IIMs development. Furthermore, recently GWAS [14] had been undertaken on European DM patients. This study showed phospholipase C–like 1 (*PLCL1*) gene, B lymphoid tyrosine kinase (*BLK*) gene, and chemokine (C-C motif) ligand 21 (*CCL21*) gene were associated with DM risk. This was the first GWAS with regard to DM, and it confirmed the *MHC* as the major genetic region associated with DM and revealed DM share enrichment of genetic loci with other autoimmune diseases.

The TNF-α–induced protein 3 (*TNFAIP3*) gene, located on chromosome 6q23, participates in nuclear factor κB *(NF-κB)* signaling pathways. It is known that genetic variants of the *TNFAIP3* gene loci were associated with susceptibility to multiple human autoimmune and inflammatory diseases including rheumatoid arthritis (RA), systemic lupus erythematous (SLE), sjogren's syndrome (SS), systemic sclerosis (SSc), psoriasis (PsA) and inflammatory bowel diseases (IBDs) [15]–[17]. Of these gene variants, two SNPs (rs2230926 and rs5029939) were mostly widely investigated, and associated with diverse rheumatic diseases [15]–[17]. Chinoy et al. [18] manifested *NF-κB*-related gene (*IKBL62*) may confer susceptibility to IIMs. The interferon-induced helicase (*IFIH1*) gene, also known as melanoma differentiation-associated 5 (*MDA5*), is located at the chromosome 2q24.3. So far, numerous of case-control studies had been conducted to assess the associations of the *IFIH1* rs1990760 and rs3747517 polymorphisms with many kinds of autoimmune diseases [19]–[22]. Gono et al. [10] reported the *IFIH1* rs1990760 “AA” genotype might be a risk factor for the onset of ILD with PM in the Japanese population. The interferon regulatory factor 5 *(IRF5)* gene is located on human chromosome 7q32 and contains nine exons. *IRF5* is fundamentally expressed in almost all lymphoid organs (except thymus), especially in B cells, monocytes plasmacytoid dendritic cells and monocyte-derived dendritic cells [23]–[24]. In recent decades, a number of studies had indicated *IRF5* gene displayed a strong association with varied autoimmune diseases [25]–[27]. The *IFIH1* gene and *IRF5* gene both participate in type I interferon (IFN) signaling pathway. Previously, the microarray studies revealed that IFN pathway was involved in the pathogenesis of DM and observed up-regulated in muscle tissue, skin tissue and peripheral blood cells [28]–[30]. The up-regulation of IFN pathway may be a more sensitive marker of disease activity in DM.

Considering the roles of *TNFAIP3*, *IFIH1*, and *IRF5* in innate and cell-mediated immunity and the reported associations with several autoimmune diseases, we hypothesized that some of the related polymorphisms of *TNFAIP3*, *IFIH1*, and *IRF5* gene might be part of the genetic background that results in the development of IIMs in a Chinese Han population.

## Methods

### Subjects

This study was designed as a large cross-sectional study, and we recruited 286 PM patients and 535 DM patients from two different sources. Between February 2013 and May 2014, 143 PM patients and 307 DM patients were enrolled from the Peking Union Medical College Hospital. Supported by the Research Special Fund for Public Welfare Industry of Health, 155 PM patients and 223 DM patients were recruited through the cooperation of three centers in China. Finally, 828 PM/DM patients were collected in our study. All patients were 18 years or older at the onset of disease and had probable or definite myositis assessed by at least two rheumatologists according to the criteria of Bohan and Peter [31]–[32]. Patients with myositis–CTD overlap syndrome were excluded if they met either the following published criteria (American College of Rheumatology (ACR) criteria for SLE [33], ACR criteria for RA [34], ACR criteria for SSc [35] and American and European consensus criteria for SS [36]) or the criteria for mixed CTD by Sharp et al [37]. And we also excluded amyopathic dermatomyositis (ADM), who could not meet the traditional criteria of Sontheimer [38]. As IBM is much less prevalent among non-Caucasian than Caucasian populations, IBM patients were not enrolled. Patients with myasthenia gravis, myasthenia syndrome, muscular dystrophy, inherited, metabolic, or infectious myopathies or muscle diseases caused by other factors were systematically excluded. 968 ethnically matched healthy controls from the Peking Union Medical College Hospital were recruited during their physical examinations according to the following rules: 1) no significant history of rheumatologic disease; 2) no family history of rheumatologic diseases; 3) normal biochemical and immunological profiles; and 4) negative serology for anti-Jo-1 and anti-Mi-2 antibodies. This study was approved by the Ethics Committee of the Peking Union Medical College Hospital, and all participants signed a written informed consent.

### Selection of SNPs

Given the dominant functions of *TNFAIP3*, *IFIH1*, and *IRF5* in the autoimmune diseases, 6 SNPs (rs2230926, rs5029939, rs1990760, rs3747517, rs4728142 and rs729302) of these genes, which had previously illustrated in a positive association with other immune-mediated diseases based on GWAS or candidate gene studies, were used for further analysis. The information of each SNP was described in Table S1 in the File S1.

### Genotyping

DNA of all patients and controls were extracted from peripheral white blood cells by using kits from Bioteke (Beijing, China) and following the manufacturer's instructions. The DNA of each participant was genotyped using Sequenom MassArray system (San Diego, CA, USA) according to the manufacturer's protocol. Primers for the multiplex polymerase chain reaction (PCR) and for locus-specific single-base extension were designed by the MassArray Assay Design 4.0 software. The PCR was carried out in a 384 plate, and the products were used for locus-specific single-base extension reactions. The final products were then desalted and transferred to a 384-element SpectroCHIP array (Sequenom, CA). Allele detection was performed by matrix-assisted laser desorption ionization–time-of-flight mass spectrometry (MALDI-TOF MS). The resultant mass spectrogram data were analyzed using MassArray Typer software.

### Statistical analysis

For the association analysis between TNFAIP3, IFIH1, and IRF5 polymorphisms and the three clinical subgroups (all PM/DM patients, PM patients and DM patients vs. control), statistical analysis was accomplished by PLINK v1.07 software (Shaun Purcell, Boston, USA) [39]. The Hardy–Weinberg equilibrium (HWE) in healthy controls was evaluated by using the Chi-square (χ^2^) test for these six SNPs. Any SNPs that deviated from the HWE (*P*<0.05 in the control groups) would be excluded from subsequent analysis. Genotype and allele distribution between patients and controls were analyzed by the χ^2^ test, and *P* values (corrected for multiple comparisons by the Bonferroni adjustment test) less than 0.05 were regarded as statistically significant. The odds ratio (OR) of associations was calculated with 95% confidence interval (95% CI). For genetic model testing (additive model, dominant model, and recessive model), genotype frequencies were further analyzed using logistic regression models. Sub-phenotype stratification analysis with regard to the association study for these six SNPs and the presence of ILD was carried out by the results of the following three comparisons: patients (all PM/DM patients, PM patients and DM patients) with ILD vs. all controls, patients without ILD vs. all controls, and patients with ILD vs. without ILD. The genetic power for this case-control study was calculated with the statistical program Genetic Power Calculator [40]. Haplotype analysis was performed by Haploview software v4.2 [41].

## Results

### Characterization of study subjects

The fundamental characteristics of all the participators were summarized in Table 1. In present study, 298 PM patients (73.6% women) and 530 DM patients (76.6% women) were enrolled. The mean ages for PM patients and DM patients were 45.6±14.9 and 46.8±15.5 years, respectively. In a word, a total of 828 adult-onset PM/DM patients (75.1% women; mean age 46.2±15.2 years) were collected. For these patients, 166 of 298 PM patients (55.7%) and 297 of 530 DM patients (56.0%) had ILD. Finally, 460 PM/DM patients had complicated with ILD and 361 patients not. The ethnically matched healthy controls included 968 subjects (83.7% women; mean age 43.1±12.6 years). The rs729302 SNP in the IRF5 gene region deviated from HWE in the control group (P_HWE_<0.05) and was excluded from further analysis. The remaining five SNPs (rs2230926, rs5029939, rs1990760, rs3747517 and rs4728142) from the three genes (*TNFAIP3*, *IFIH1*, and *IRF5*) were in HWE and produced an average genotyping call rate of more than 96%. The accuracy was 100% as 60 samples were duplicated genotyped and the consequences were consistent. The power analysis revealed that our sample size had more than 80% power (α = 0.05) for detecting association with an OR of 1.10–1.60 for both heterozygotes and homozygotes.

**Table 1:** Clinical data for PM/DM patients and controls.

Characteristic	Patients	Controls
Number of subjects (DM/PM)	828(530/298)	968
Female ratio (%)	83.7	75.1
Average age	46.2±15.2	43.1±12.6
DM with ILD, No/total (%)	297/530(56.0)	-
PM with ILD, No/total (%)	166/298(55.7)	-

PM: polymyositis; DM: dermatomyositis; ILD: interstitial lung disease.

### Association of these SNPs with PM/DM in the Han population

Table 2 summarized the genotype and allele frequencies for these five SNPs (rs2230926, rs5029939, rs1990760, rs3747517 and rs4728142). For the *TNFAIP3* region, the rs2230926 allele and genotype were associated with PM patients or PM/DM patients (*P_c_* = 4.0×10^−3^ and *P_c_* = 0.02; *P_c_* = 7.5×10^−3^ and *P_c_* = 0.04, respectively). And rs5029939 showed a significant association with PM patients or PM/DM patients (*P_c_* = 5.5×10^−3^ and *P_c_* = 6.0×10^−3^, respectively) when allele frequencies were analyzed. For the *IFIH1* gene region, neither of the two SNPs (rs1990760 and rs3747517) demonstrated significant differences in allele or genotype frequencies between patients and controls (all, *P_c_*>0.05). For the *IRF5* gene, the genotype frequencies of rs4728142 manifested associations with DM patients or PM/DM patients (*P_c_* = 0.042 and *P_c_* = 0.048, respectively). Additionally, the percentage of PM/DM patients with A allele of rs4728142 was significantly higher than that in the healthy controls (*P_c_* = 0.026).

**Table 2:** Allele and genotype distribution of the *TNFAIP3*, *IRF5*, *IFIH1* gene markers in PM/DM patients and controls.

			Allele (%)					Genotype (%)					
Gene	SNPs	Groups	G	T	OR (95%CI)	*P*	*P_c_*	GG	GT	TT	?^2^	*P*	*P_c_*
TNFAIP3	rs2230926	DM	63(6.1)	967(93.9)	1.45(1.04–2.03)	0.03	0.15	4(0.8)	55(10.7)	456(88.5)	NA*	0.06	0.30
		PM	45(7.8)	533(92.2)	1.88(1.30–2.74)	8.0×10^−4^	4.0×10^−3^	2(0.7)	41(14.2)	246(85.1)	NA*	4.0×10^−3^	0.02
		DM+PM	108(6.7)	1500(93.3)	1.61(1.20–2.16)	1.5×10^−3^	7.5×10^−3^	6(0.7)	96(12.0)	702(87.3)	NA*	7.7×10^−3^	0.04
		Controls	83(4.3)	1851(95.7)				1(0.1)	81(8.4)	885(91.5)			
			G	C				GG	GC	CC			
	rs5029939	DM	62(6.0)	968(94.0)	1.50(1.07–2.11)	0.02	0.10	3(0.6)	56(10.9)	456(88.5)	NA*	0.22	1.10
		PM	43(7.4)	537(92.6)	1.88(1.28–2.76)	1.1×10^−3^	5.5×10^−3^	0(0.0)	43(14.8)	247(85.2)	NA#	NA#	NA#
		DM+PM	105(6.5)	1505(93.5)	1.64(1.21–2.21)	1.2×10^−3^	6.0×10^−3^	3(0.4)	99(12.3)	703(87.3)	NA*	0.016	0.08
		Controls	79(4.1)	1853(95.9)				0(0.0)	79(8.2)	887(91.8)			
			T	C				TT	TC	CC			
IFIH1	rs1990760	DM	226(21.9)	808(78.1)	1.04(0.87–1.25)	0.67	3.35	31(6.0)	164(31.7)	322(62.3)	1.65	0.44	2.20
		PM	136(23.4)	446(76.6)	1.14(0.91–1.42)	0.26	1.30	18(6.2)	100(34.4)	173(59.4)	1.58	0.45	2.25
		DM+PM	362(22.4)	1254(77.6)	1.07(0.92–1.26)	0.38	1.90	49(6.1)	264(32.6)	495(61.3)	2.05	0.36	1.80
		Controls	410(21.2)	1526(78.8)				44(4.5)	322(33.3)	602(62.2)			
			G	A				GG	GA	AA			
	rs3747517	DM	338(33.0)	686(67.0)	0.98(0.83–1.15)	0.78	3.90	52(10.2)	234(45.7)	226(44.1)	0.61	0.74	3.70
		PM	198(34.5)	376(65.5)	1.05(0.86–1.27)	0.66	3.30	30(10.4)	138(48.1)	119(41.5)	1.31	0.52	2.60
		DM+PM	536(33.5)	1062(66.5)	1.00(0.87–1.15)	0.98	4.90	82(10.3)	372(46.5)	345(43.2)	1.15	0.56	2.80
		Controls	648(33.5)	1286(66.5)				110(11.4)	428(44.3)	429(44.3)			
			A	G				AA	AG	GG			
IRF5	rs4728142	DM	179(17.2)	863(82.8)	1.30(1.05–1.59)	0.01	0.05	8(1.5)	163(31.3)	350(67.2)	9.56	8.4×10^−3^	0.042
		PM	98(17.3)	470(82.7)	1.30(1.01–1.68)	0.04	0.20	9(3.2)	80(28.2)	195(68.6)	4.30	0.12	0.60
		DM+PM	277(17.2)	1333(82.8)	1.30(1.08–1.56)	5.2×10^−3^	0.026	17(2.1)	243(30.2)	545(67.7)	9.30	9.6×10^−3^	0.048
		Controls	267(13.8)	1667(86.2)				18(1.9)	231(23.9)	718(74.2)			

PM: polymyositis; DM: dermatomyositis; OR: odds ratio; CI: confidence interval; χ^2^: Chi-square test; *P_c_*: *P* value corrected by Bonferroni method; NA: not available;

*: the P value of genotypic analysis was calculated under the logistic regression analysis;

# : This research's result demonstrated that rs5029939 GG genotype in PM patients was 0. We failed to calculate its genotypic frequency.

Further logistic regression analysis was performed based upon three genetic models (additive, dominant, and recessive model). The analysis outcomes of these three models were summarized in Table 3. In the additive and the dominant model, significant associations were observed in PM patients or PM/DM patients for two SNPs (rs2230926 and rs5029939) in *TNFAIP3* gene region (all, *P_c_*<0.05). None of the three genetic models showed any significant differences between cases and controls for two SNPs (rs1990760 and rs3747517) of *IFIH1* gene (all, *P_c_*>0.05). For rs4728142 in *IRF5* gene region, associations were also observed under the additive and the dominant model in PM/DM patients (all, *P_c_*<0.05). In additional, rs4728142 indicated weak association with DM patients in the additive model.

**Table 3:** Analysis of the five SNPs based on three genetic models.

			Additive model		Dominant model		Recessive model	
Gene	SNPs	Group	*P_c_*	OR (95%CI)	*P_c_*	OR (95%CI)	*P_c_*	OR (95%CI)
TNFAIP3	rs2230926	DM	0.16	1.44(1.03–2.02)	0.32	1.40(0.98–2.00)	0.35	7.56(0.84–67.8)
		PM	4.8×10^−3^	1.89(1.30–2.77)	8.2×10^−3^	1.89(1.27–2.80)	0.60	6.73(0.61–74.5)
		DM+PM	9.1×10^−3^	1.59(1.19–2.13)	0.02	1.57(1.15–2.13)	0.33	7.26(0.87–60.5)
	rs5029939	DM	0.10	1.51(1.07–2.12)	0.20	1.45(1.02–2.07)	NA#	NA#
		PM	4.7×10^−3^	2.00(1.31–2.91)	4.7×10^−3^	2.00(1.31–2.91)	NA#	NA#
		DM+PM	5.7×10^−3^	1.66(1.22–2.24)	0.01	1.63(1.20–2.22)	NA#	NA#
IFIH1	rs1990760	DM	3.36	1.04(0.87–1.25)	4.86	1.00(0.80–1.24)	1.13	1.34(0.84–2.15)
		PM	1.32	1.13(0.91–1.41)	2.00	1.12(0.86–1.47)	1.29	1.39(0.79–2.44)
		DM+PM	1.93	1.07(0.92–1.26)	3.44	1.04(0.86–1.26)	0.77	1.36(0.89–2.06)
	rs3747517	DM	3.92	0.98(0.83–1.15)	4.67	1.01(0.81–1.25)	2.38	0.88(0.62–1.25)
		PM	3.29	1.05(0.86–1.27)	1.92	1.13(0.86–1.47)	3.32	0.91(0.59–1.39)
		DM+PM	4.91	1.00(0.87–1.15)	3.09	1.05(0.87–1.27)	2.27	0.89(0.66–1.21)
IRF5	rs4728142	DM	0.06	1.31(1.06–1.62)	0.02	1.41(1.12–1.78)	3.24	0.82(0.36–1.90)
		PM	0.21	1.30(1.01–1.68)	0.31	1.32(0.99–1.76)	0.94	1.73(0.77–3.88)
		DM+PM	0.02	1.31(1.09–1.58)	0.01	1.38(1.12–1.69)	3.53	1.14(0.58–2.22)

PM: polymyositis; DM: dermatomyositis; OR odds ratio; CI confidence interval; *P_c_*: *P* value corrected by Bonferroni method; NA: not available.

# : The GG genotype frequencies of rs5029939 were too low to carry out recessive genetic model analysis.

### Association between TNFAIP3, IFIH1, and IRF5 polymorphisms and the ILD phenotype of PM/DM

Then, it had also been performed that the association analysis between *TNFAIP3*, *IFIH1*, and *IRF5* polymorphisms and ILD phenotype of PM/DM patients. The associations between these five SNPs and PM/DM patients with/without ILD were summarized in Table 4. Significantly, rs5029939 in *TNFAIP3* gene was associated with PM patients or PM/DM patients with ILD involvement (*P_c_* = 0.03 and *P_c_* = 0.02, respectively). Similarly, there was a statistically significant difference in rs2230926 between PM patients or PM/DM patients with ILD and healthy controls (*P_c_* = 0.016 and *P_c_* = 0.04, respectively). However, rs1990760, rs3747517 in *IFIH1* gene and rs4728142 in *IRF5* gene region were not statistically significant associated with PM/DM patients with/without ILD in present study.

**Table 4:** Association between the five SNPs and PM/DM with ILD.

		rs2230926(*TNFAIP3*)		rs5029939(*TNFAIP3*)		rs1990760(*IFIH1*)		rs3747517(*IFIH1*)		rs4728142(*IRF5*)	
Disease	Group	*P_c_*	OR (95%CI)	*P_c_*	OR (95%CI)	*P_c_*	OR (95%CI)	*P_c_*	OR (95%CI)	*P_c_*	OR (95%CI)
DM	P vs. N	3.14	0.88(0.53–1.47)	4.11	1.06(0.63–1.79)	0.53	0.78(0.58–1.05)	4.00	1.04(0.80–1.35)	2.51	0.90(0.65–1.24)
	P vs. C	0.64	1.37(0.91–2.07)	1.77	1.54(1.03–2.31)	2.76	0.93(0.74–1.17)	4.70	0.99(0.82–1.21)	0.51	1.23(0.96–1.59)
	N vs. C	0.22	1.56(1.01–2.41)	0.53	1.45(0.92–2.29)	0.79	1.19(0.93–1.52)	3.55	0.96(0.77–1.20)	0.15	1.38(1.05–1.81)
PM	P vs. N	3.86	1.10(0.59–2.03)	4.65	1.03(0.55–1.92)	3.30	0.92(0.62–1.35)	3.82	0.95(0.67–1.34)	2.64	1.15(0.74–1.79)
	P vs. C	0.016	1.96(1.24–3.10)	0.03	1.90(1.19–3.05)	2.73	1.09(0.82–1.45)	4.37	1.02(0.79–1.31)	0.21	1.39(1.01–1.90)
	N vs. C	0.13	1.79(1.07–3.00)	0.09	1.85(1.10–3.11)	1.30	1.19(0.88–1.61)	3.00	1.08(0.82–1.41)	1.57	1.20(0.84–1.72)
DM+PM	P vs. N	4.22	0.96(0.65–1.42)	4.16	1.04(0.70–1.56)	0.60	0.83(0.66–1.05)	4.95	1.00(0.81–1.24)	4.35	0.98(0.75–1.27)
	P vs. C	0.04	1.58(1.12–2.22)	0.02	1.67(1.18–2.36)	4.5	0.99(0.81–2.00)	4.90	1.00(0.85–1.19)	0.11	1.29(1.04–1.60)
	N vs. C	0.04	1.64(1.14–2.37)	0.07	1.60(1.10–2.33)	0.47	1.19(0.97–1.46)	4.96	1.00(0.83–1.20)	0.11	1.31(1.04–1.66)

DM: dematomysitis; PM: polymyositis; ILD: interstitial lung disease; Group P: patients with ILD; Group N: patients without ILD; Group C: Healthy controls; *P_c_*: *P* value corrected by Bonferroni method. Group P (DM: n = 297; PM: n = 166; DM+PM: n = 463); Group N (DM: n = 233; PM: n = 132; DM+PM: n = 365); Group C (n = 968).

### Haplotype analysis of TNFAIP3 SNPs and patients

**Figure 1:**
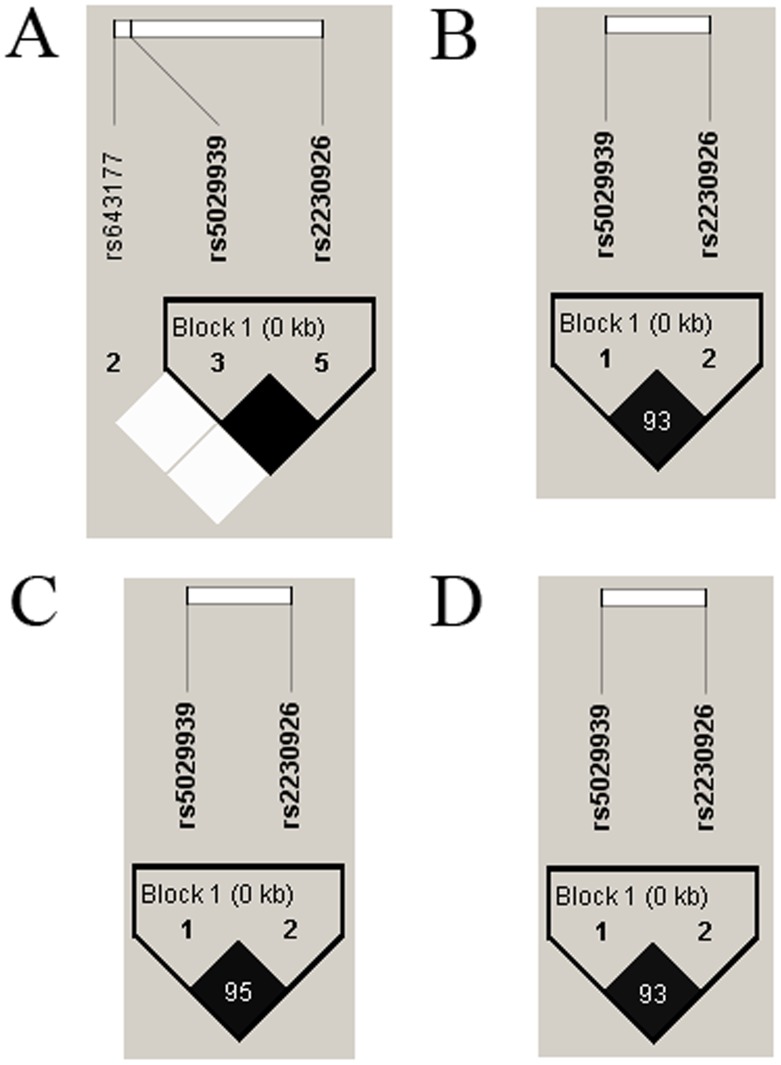
Linkage disequilibrium (LD) analysis of the SNPs in the *TNFAIP3* gene region. The LD plots were generated by Haploview software v4.2 and data from our study were similar to that from the HapMap CHB population. The number (divided by 100) in the small square represents r^2^ value and ranges from 0 to 1. The two SNPs (rs2230926 and rs5029939) in *TNFAIP3* reside in an LD block. (A): The data from HapMap CHB. B: The data analysis between DM patients and healthy controls from our study. C: The data analysis between PM patients and healthy controls from our study. D: The data analysis between PM/DM patients and healthy controls from our study.

We used Haploview software to further analyze the distributions of the haplotypes in the *TNFAIP3* SNPs between patients and healthy controls. The results from the LD analysis of the SNPs (rs2230926, rs5029939) in our study and the data from the HapMap CHB population were shown in Table 5 and Fig. 1. Data from HapMap CHB and the present study illustrated no significant differences. And strong LD association existed between rs2230926 and rs5029939 (r^2^ = 1). The CT haplotype (rs2230926 C–rs5029939 T) had a lower frequency between DM, PM or PM/DM patients and controls (*P_c_* = 0.04, *P_c_* = 2.5×10^−3^ and *P_c_* = 2.0×10^−3^, respectively)(Table 5).

**Table 5:** Haplotype analysis of *TNFAIP3* SNPs between patients and controls.

	Haplotypes						
Groups	rs2230926	rs5029939	Total of frequency	Case	Control	?^2^	*P_c_*
DM	C	T	0.95	0.94	0.96	6.73	0.04
	G	G	0.05	0.06	0.04	5.57	0.09
PM	C	T	0.95	0.92	0.96	12.0	2.5×10^−3^
	G	G	0.05	0.08	0.04	10.2	7×10^−3^
DM+PM	C	T	0.95	0.93	0.96	12.3	2.0×10^−3^
	G	G	0.05	0.07	0.04	10.4	6.5×10^−3^

PM: polymyositis; DM: dermatomyositis; χ^2^: Chi-square test; *P_c_*: *P* value corrected by Bonferroni method.

## Discussion

The sample size of present study was the largest candidate gene association study executed in PM/DM to date and the first one to investigate the association of *TNFAIP3*, *IFIH1*, and *IRF5* polymorphisms with PM/DM in Chinese Han population [5]–[13]. We intend to examine the genetic contribution of *TNFAIP3*, *IFIH1*, and *IRF5* to PM/DM based upon the postulated roles of each of these genes' products in innate and cell-mediated immunity in PM/DM and their described associations with autoimmune diseases. Significantly, our study confirmed *TNFAIP3* and *IRF5* gene polymorphisms were associated with PM/DM patients or these patients with ILD, and indicated that *TNFAIP3* and *IRF5* might be the susceptibility gene for PM/DM patients in Chinese Han population. The results were consistent with the previous findings undertaken on other auto-inflammation diseases with regard to *TNFAIP3* and *IRF5* gene polymorphisms [15]–[17], [25]–[27].

The *TNFAIP3* gene, encoded ubiquitin-modifying enzyme known as A20, inhibits the activation of *NF-κB* signaling pathways, including these *TNF* and Toll-like receptors [42]. The deficiencies of A20 expression are related with the development of various human autoimmune disorders [43]. The immune responses in A20-deficient mice present severe multi-organ inflammation, damage to joints, and finally develop autoimmunity [44]. Furthermore, the mice, which lacked A20 in myeloid cells spontaneously, finally turn into RA with many features such as severe destructive polyarthritis [45]. In addition, Deficiency of A20 in B cells results in inflammation and leads to autoimmune response in old mice [46]. Previously, plenty of GWAS and candidate gene association studies suggested *TNFAIP3* gene loci rs2230926 and rs5029939 were associated with diverse rheumatic diseases [15]–[17]. Studies denoted that DM could be overlapped with SSc [47], SLE [48] and other connective-tissue disease. In the current study of the Han Chinese population, *TNFAIP3* SNPs (rs2230926 and rs5029939) illustrated significant association with PM patients or PM/DM patients and these patients with ILD. Thus, rs2230926 and rs5029939 may in fact play a dominant role in the pathogenesis of multiple autoimmune diseases as well as to PM/DM. It is also worthwhile to note that in the present study, the GG genotype frequencies of rs2230926 and rs5029939 were too low to carry out genotypic analysis and recessive genetic model analysis. The P value of genotypic analysis was calculated under the logistic regression analysis. Future studies about PM/DM patients using larger sample sizes should be performed to confirm these outcomes.

The *IFIH1* gene is a member of the retinoic acid-inducible gene I-like helicase (RLH) family [49]–[50], and *IFIH1* gene encodes a viral RNA- activated apoptosis protein, which is an early IFN beta responsive protein [50]. Walsh et al. [51] demonstrated that IFNα/β-inducible genes (such as *IFIH1* gene) were the greatest highly overexpressed genes in patients with active DM and patients with PM, but not in healthy controls. And the up-regulation of the IFN protein signature had increased additional markers of disease activity and insight into the pathogenesis of PM/DM. Until now, a large number of studies indicated that *IFIH1* polymorphisms showed susceptibility to multiple autoimmune disorders [19]–[22]. Recently, an investigation, regarding *IFIH1* rs1990760 associated with susceptibility to SLE and PM/DM, was performed in the Japanese population, which suggested *IFIH1* rs1990760 polymorphism was not significantly associated with PM/DM as a whole in this study, but only showed the AA genotype tended to be found with higher frequency in the PM with ILD subset. Similarly, our current study carried out in the Chinese population didn't manifest the positive associations between *IFIH1* polymorphisms and PM/DM patients or these patients with ILD. The SNPs (rs1990760 and rs3747517) in *IFIH1* gene chose in our study were those with the strongest associations with other autoimmune disease in the Chinese Han population. As the SNPs in our study did not illustrate positive associations with PM/DM, future studies might evaluate whether other SNPs in *IFIH1* gene are associated with susceptibility to PM/DM.

The *IRF5* gene is a part of the transcription factor IRF family, which contains nine transcription factors. This gene encodes IRF protein with diverse roles, including virus-mediated activation of interferon, and modulation of cell growth, differentiation, apoptosis, and immune system activity. When viruses infect, expression of *IRF5* is up-regulated by *IFN-α*, and subsequently *IRF5* up-regulates IFN-inducible genes, comprising pro-inflammatory cytokines such as *IL-10*, and also those participated in apoptosis and the early immune response [52]. Therefore, this up-regulation of important molecules in the IFN signaling pathway may have prominent functional influence on the pathogenesis of autoimmune diseases. Thence, numerous of studies found IRF5 gene polymorphisms were associated with susceptibility to multiple autoimmune disorders [25]–[27]. Significantly, this present candidate gene association studies regarding *IRF5* polymorphism associated with PM/DM risk demonstrated positive results. And the consequences were consistent with the previous findings undertaken on other auto-inflammation diseases. Although we excluded rs729302 because of its departure from HWE in the control group, the correlation of this SNP to PM/DM was seen without consideration of HWE (data not shown), implying a possible association between rs729302 and PM/DM in Han Chinese population.

Because IIMs are a group of rare autoimmune disease, difficulties always were encountered in previous genetic association studies when investigator recruited an adequate number of patients for analysis in studies that examined SNPs with an appropriate sample size. Given the above limitations, our study firstly enrolled the largest number of PM/DM patients in Chinese Han population, who fulfilled the international guidelines [31]–[32]. Therefore, the present investigation had sufficient statistical power (more than 80%) to examine moderate or even marginal associations. It is a remarkable fact that our study found significantly positive associations between *TNFAIP3* (rs2230926 and rs5029939), *IRF5* (rs4728142) gene polymorphisms and PM/DM or these patients with ILD. None of these SNPs was previously reported to be associated with PM/DM or these patients with ILD. Therefore, these SNPs may play a potential role in the pathogenesis of PM/DM or these patients with ILD. In addition, our study failed to analyze the potential association of these genetic variants with some special clinical subtypes of PM/DM in this population, such as serological phenotypes (autoantibody profiles).

In summary, our present study was the first investigation to indicate that *TNFAIP3* and *IRF5* might be the susceptibility gene for PM/DM patients in Chinese Han population. Although the present study is the largest candidate gene association study performed to date for PM/DM, it is still limited and more research is required to understand the associations of *TNFAIP3*, *IFIH1*, and *IRF5* with PM/DM in different ethnic populations. In the Chinese population, it isn't been reported about GWAS on PM/DM, in order to better understand the pathogenesis of PM/DM, we are looking forward to conduct the GWAS on PM/DM in the Chinese population.

## Supporting Information

Table S1 The detailed information of SNPs in this study.
(DOC)
