# Supplementary material for: Genetic Association Study of TNFAIP3, IFIH1, IRF5 Polymorphisms with Polymyositis/Dermatomyositis in Chinese Han Population
Source: PLoS One. 2014 Oct 22;9(10):e110044. doi: 10.1371/journal.pone.0110044 (PMC4206287; doi:10.1371/journal.pone.0110044)
Supplement: Table S1 — The detailed information of SNPs in this study. (DOC) [file pone.0110044.s001.doc]

**Table S1.** The detail information of SNPs in this study.

| **Gene** | **SNPs** | **Position (NCBI)** | **Primary SNP disease source** |
| --- | --- | --- | --- |
| TNFAIP3 | rs2230926 | chr6: 137874929 | SLE [1-13], RA [4,14-19], SS [15,20], SSc [21], PsA [15,22-23], CD [15] |
|  | rs5029939 | chr6: 137874586 | SLE [6,8-9,11,24], SS [25], SSc [20,26], PsA [21] |
| IFIH1 | rs1990760 | chr2: 162267541 | T1D [27-37], SLE [37-44], GD [37,45-48], MS [31,37,49], RA [37,50-51], HT [37,47-48], AAD [37,45,47], PM/DM [43], PsA [52] |
|  | rs3747517 | chr2: 162272314 | T1D [32-33,35], MS [48], PsA [52] |
| IRF5 | rs4728142 | chr7: 128933913 | SLE [5,53-58], SSc [59-62], MS [53,63-64], IBD [65-66], UC [66-67], CD [66] |
|  | rs729302 | chr7: 128928906 | SLE [5,55,68-72], RA [73-82], JIA [83], MS [63] |

**References:**

1. Musone SL, Taylor KE, Lu TT, Nititham J, Ferreira RC, et al. (2008) Multiple polymorphisms in the TNFAIP3 region are independently associated with systemic lupus erythematosus. Nat Genet 40: 1062-1064.

2. Graham RR, Cotsapas C, Davies L, Hackett R, Lessard CJ, et al. (2008) Genetic variants near TNFAIP3 on 6q23 are associated with systemic lupus erythematosus. Nat Genet 40: 1059-1061.

3. Adrianto I, Wen F, Templeton A, Wiley G, King JB, et al. (2011) Association of a functional variant downstream of TNFAIP3 with systemic lupus erythematosus. Nat Genet 43: 253-258.

4. Shimane K, Kochi Y, Horita T, Ikari K, Amano H, et al. (2010) The association of a nonsynonymous single-nucleotide polymorphism in TNFAIP3 with systemic lupus erythematosus and rheumatoid arthritis in the Japanese population. Arthritis Rheum 62: 574-579.

5. Yang W, Shen N, Ye DQ, Liu Q, Zhang Y, et al. (2010) Genome-wide association study in Asian populations identifies variants in ETS1 and WDFY4 associated with systemic lupus erythematosus. PLoS Genet 6: e1000841.

6. Bates JS, Lessard CJ, Leon JM, Nguyen T, Battiest LJ, et al. (2009) Meta-analysis and imputation identifies a 109 kb risk haplotype spanning TNFAIP3 associated with lupus nephritis and hematologic manifestations. Genes Immun 10: 470-477.

7. Zhong H, Li XL, Li M, Hao LX, Chen RW, et al. (2011) Replicated associations of TNFAIP3, TNIP1 and ETS1 with systemic lupus erythematosus in a southwestern Chinese population. Arthritis Res Ther 13: R186.

8. Kim SK, Choe JY, Bae J, Chae SC, Park DJ, et al. (2014) TNFAIP3 gene polymorphisms associated with differential susceptibility to rheumatoid arthritis and systemic lupus erythematosus in the Korean population. Rheumatology (Oxford).

9. Kadota K, Mori M, Yanagimachi M, Miyamae T, Hara T, et al. (2013) Analysis of gender differences in genetic risk: association of TNFAIP3 polymorphism with male childhood-onset systemic lupus erythematosus in the Japanese population. PLoS One 8: e72551.

10. Lee YH, Song GG (2012) Associations between TNFAIP3 gene polymorphisms and systemic lupus erythematosus: a meta-analysis. Genet Test Mol Biomarkers 16: 1105-1110.

11. Cai LQ, Wang ZX, Lu WS, Han JW, Sun LD, et al. (2010) A single-nucleotide polymorphism of the TNFAIP3 gene is associated with systemic lupus erythematosus in Chinese Han population. Mol Biol Rep 37: 389-394.

12. Kawasaki A, Ito I, Ito S, Hayashi T, Goto D, et al. (2010) Association of TNFAIP3 polymorphism with susceptibility to systemic lupus erythematosus in a Japanese population. J Biomed Biotechnol 2010: 207578.

13. Fan Y, Tao JH, Zhang LP, Li LH, Ye DQ (2011) The association between BANK1 and TNFAIP3 gene polymorphisms and systemic lupus erythematosus: a meta-analysis. Int J Immunogenet 38: 151-159.

14. Orozco G, Hinks A, Eyre S, Ke X, Gibbons LJ, et al. (2009) Combined effects of three independent SNPs greatly increase the risk estimate for RA at 6q23. Hum Mol Genet 18: 2693-2699.

15. Musone SL, Taylor KE, Nititham J, Chu C, Poon A, et al. (2011) Sequencing of TNFAIP3 and association of variants with multiple autoimmune diseases. Genes Immun 12: 176-182.

16. Maxwell JR, Gowers IR, Wilson AG (2009) Complex genetic association of 6q23 with autoimmune rheumatic conditions. Arthritis Res Ther 11: 107.

17. Lee YH, Bae SC, Choi SJ, Ji JD, Song GG (2012) Associations between TNFAIP3 gene polymorphisms and rheumatoid arthritis: a meta-analysis. Inflamm Res 61: 635-641.

18. Song GG, Bae SC, Lee YH (2013) Pathway analysis of genome-wide association studies on rheumatoid arthritis. Clin Exp Rheumatol 31: 566-574.

19. Zhang X, Li W, Zhang X, Zhao L, Zhang X, et al. (2014) Single nucleotide polymorphisms in TNFAIP3 were associated with the risks of rheumatoid arthritis in northern Chinese Han population. BMC Med Genet 15: 56.

20. Nocturne G, Boudaoud S, Miceli-Richard C, Viengchareun S, Lazure T, et al. (2013) Germline and somatic genetic variations of TNFAIP3 in lymphoma complicating primary Sjogren's syndrome. Blood 122: 4068-4076.

21. Koumakis E, Giraud M, Dieude P, Cohignac V, Cuomo G, et al. (2012) Brief report: candidate gene study in systemic sclerosis identifies a rare and functional variant of the TNFAIP3 locus as a risk factor for polyautoimmunity. Arthritis Rheum 64: 2746-2752.

22. Nair RP, Duffin KC, Helms C, Ding J, Stuart PE, et al. (2009) Genome-wide scan reveals association of psoriasis with IL-23 and NF-kappaB pathways. Nat Genet 41: 199-204.

23. Tejasvi T, Stuart PE, Chandran V, Voorhees JJ, Gladman DD, et al. (2012) TNFAIP3 gene polymorphisms are associated with response to TNF blockade in psoriasis. J Invest Dermatol 132: 593-600.

24. Lodolce JP, Kolodziej LE, Rhee L, Kariuki SN, Franek BS, et al. (2010) African-derived genetic polymorphisms in TNFAIP3 mediate risk for autoimmunity. J Immunol 184: 7001-7009.

25. Li Y, Zhang K, Chen H, Sun F, Xu J, et al. (2013) A genome-wide association study in Han Chinese identifies a susceptibility locus for primary Sjogren's syndrome at 7q11.23. Nat Genet 45: 1361-1365.

26. Dieude P, Guedj M, Wipff J, Ruiz B, Riemekasten G, et al. (2010) Association of the TNFAIP3 rs5029939 variant with systemic sclerosis in the European Caucasian population. Ann Rheum Dis 69: 1958-1964.

27. Smyth DJ, Cooper JD, Bailey R, Field S, Burren O, et al. (2006) A genome-wide association study of nonsynonymous SNPs identifies a type 1 diabetes locus in the interferon-induced helicase (IFIH1) region. Nat Genet 38: 617-619.

28. Barrett JC, Clayton DG, Concannon P, Akolkar B, Cooper JD, et al. (2009) Genome-wide association study and meta-analysis find that over 40 loci affect risk of type 1 diabetes. Nat Genet 41: 703-707.

29. Todd JA, Walker NM, Cooper JD, Smyth DJ, Downes K, et al. (2007) Robust associations of four new chromosome regions from genome-wide analyses of type 1 diabetes. Nat Genet 39: 857-864.

30. Plagnol V, Howson JM, Smyth DJ, Walker N, Hafler JP, et al. (2011) Genome-wide association analysis of autoantibody positivity in type 1 diabetes cases. PLoS Genet 7: e1002216.

31. Martinez A, Santiago JL, Cenit MC, de Las HV, de la Calle H, et al. (2008) IFIH1-GCA-KCNH7 locus: influence on multiple sclerosis risk. Eur J Hum Genet 16: 861-864.

32. Liu S, Wang H, Jin Y, Podolsky R, Reddy MV, et al. (2009) IFIH1 polymorphisms are significantly associated with type 1 diabetes and IFIH1 gene expression in peripheral blood mononuclear cells. Hum Mol Genet 18: 358-365.

33. Nejentsev S, Walker N, Riches D, Egholm M, Todd JA (2009) Rare variants of IFIH1, a gene implicated in antiviral responses, protect against type 1 diabetes. Science 324: 387-389.

34. Jermendy A, Szatmari I, Laine AP, Lukacs K, Horvath KH, et al. (2010) The interferon-induced helicase IFIH1 Ala946Thr polymorphism is associated with type 1 diabetes in both the high-incidence Finnish and the medium-incidence Hungarian populations. Diabetologia 53: 98-102.

35. Yang H, Wang Z, Xu K, Gu R, Chen H, et al. (2012) IFIH1 gene polymorphisms in type 1 diabetes: genetic association analysis and genotype-phenotype correlation in Chinese Han population. Autoimmunity 45: 226-232.

36. Yamashita H, Awata T, Kawasaki E, Ikegami H, Tanaka S, et al. (2011) Analysis of the HLA and non-HLA susceptibility loci in Japanese type 1 diabetes. Diabetes Metab Res Rev 27: 844-848.

37. Cen H, Wang W, Leng RX, Wang TY, Pan HF, et al. (2013) Association of IFIH1 rs1990760 polymorphism with susceptibility to autoimmune diseases: a meta-analysis. Autoimmunity 46: 455-462.

38. Gateva V, Sandling JK, Hom G, Taylor KE, Chung SA, et al. (2009) A large-scale replication study identifies TNIP1, PRDM1, JAZF1, UHRF1BP1 and IL10 as risk loci for systemic lupus erythematosus. Nat Genet 41: 1228-1233.

39. Cunninghame GD, Morris DL, Bhangale TR, Criswell LA, Syvanen AC, et al. (2011) Association of NCF2, IKZF1, IRF8, IFIH1, and TYK2 with systemic lupus erythematosus. PLoS Genet 7: e1002341.

40. Harley JB, Alarcon-Riquelme ME, Criswell LA, Jacob CO, Kimberly RP, et al. (2008) Genome-wide association scan in women with systemic lupus erythematosus identifies susceptibility variants in ITGAM, PXK, KIAA1542 and other loci. Nat Genet 40: 204-210.

41. Hom G, Graham RR, Modrek B, Taylor KE, Ortmann W, et al. (2008) Association of systemic lupus erythematosus with C8orf13-BLK and ITGAM-ITGAX. N Engl J Med 358: 900-909.

42. Gateva V, Sandling JK, Hom G, Taylor KE, Chung SA, et al. (2009) A large-scale replication study identifies TNIP1, PRDM1, JAZF1, UHRF1BP1 and IL10 as risk loci for systemic lupus erythematosus. Nat Genet 41: 1228-1233.

43. Gono T, Kawaguchi Y, Sugiura T, Furuya T, Kawamoto M, et al. (2010) Interferon-induced helicase (IFIH1) polymorphism with systemic lupus erythematosus and dermatomyositis/polymyositis. Mod Rheumatol 20: 466-470.

44. Cunninghame GD, Morris DL, Bhangale TR, Criswell LA, Syvanen AC, et al. (2011) Association of NCF2, IKZF1, IRF8, IFIH1, and TYK2 with systemic lupus erythematosus. PLoS Genet 7: e1002341.

45. Sutherland A, Davies J, Owen CJ, Vaikkakara S, Walker C, et al. (2007) Genomic polymorphism at the interferon-induced helicase (IFIH1) locus contributes to Graves' disease susceptibility. J Clin Endocrinol Metab 92: 3338-3341.

46. Zhao ZF, Cui B, Chen HY, Wang S, Li I, et al. (2007) The A946T polymorphism in the interferon induced helicase gene does not confer susceptibility to Graves' disease in Chinese population. Endocrine 32: 143-147.

47. Penna-Martinez M, Ramos-Lopez E, Robbers I, Kahles H, Hahner S, et al. (2009) The rs1990760 polymorphism within the IFIH1 locus is not associated with Graves' disease, Hashimoto's thyroiditis and Addison's disease. BMC Med Genet 10: 126.

48. Ban Y, Tozaki T, Taniyama M, Nakano Y, Ban Y, et al. (2010) Genomic polymorphism in the interferon-induced helicase (IFIH1) gene does not confer susceptibility to autoimmune thyroid disease in the Japanese population. Horm Metab Res 42: 70-72.

49. Enevold C, Oturai AB, Sorensen PS, Ryder LP, Koch-Henriksen N, et al. (2009) Multiple sclerosis and polymorphisms of innate pattern recognition receptors TLR1-10, NOD1-2, DDX58, and IFIH1. J Neuroimmunol 212: 125-131.

50. Marinou I, Montgomery DS, Dickson MC, Binks MH, Moore DJ, et al. (2007) The interferon induced with helicase domain 1 A946T polymorphism is not associated with rheumatoid arthritis. Arthritis Res Ther 9: R40.

51. Martinez A, Varade J, Lamas JR, Fernandez-Arquero M, Jover JA, et al. (2008) Association of the IFIH1-GCA-KCNH7 chromosomal region with rheumatoid arthritis. Ann Rheum Dis 67: 137-138.

52. Chen G, Zhou D, Zhang Z, Kan M, Zhang D, et al. (2012) Genetic variants in IFIH1 play opposite roles in the pathogenesis of psoriasis and chronic periodontitis. Int J Immunogenet 39: 137-143.

53. Richman IB, Taylor KE, Chung SA, Trupin L, Petri M, et al. (2012) European genetic ancestry is associated with a decreased risk of lupus nephritis. Arthritis Rheum 64: 3374-3382.

54. Cunninghame GD, Manku H, Wagner S, Reid J, Timms K, et al. (2007) Association of IRF5 in UK SLE families identifies a variant involved in polyadenylation. Hum Mol Genet 16: 579-591.

55. Graham RR, Kyogoku C, Sigurdsson S, Vlasova IA, Davies LR, et al. (2007) Three functional variants of IFN regulatory factor 5 (IRF5) define risk and protective haplotypes for human lupus. Proc Natl Acad Sci U S A 104: 6758-6763.

56. Deng FY, Lei SF, Zhang YH, Zhang ZL, Guo YF (2013) Functional relevance for associations between genetic variants and systemic lupus erythematosus. PLoS One 8: e53037.

57. Armstrong DL, Zidovetzki R, Alarcon-Riquelme ME, Tsao BP, Criswell LA, et al. (2014) GWAS identifies novel SLE susceptibility genes and explains the association of the HLA region. Genes Immun.

58. Armstrong DL, Reiff A, Myones BL, Quismorio FJ, Klein-Gitelman M, et al. (2009) Identification of new SLE-associated genes with a two-step Bayesian study design. Genes Immun 10: 446-456.

59. Sharif R, Mayes MD, Tan FK, Gorlova OY, Hummers LK, et al. (2012) IRF5 polymorphism predicts prognosis in patients with systemic sclerosis. Ann Rheum Dis 71: 1197-1202.

60. Radstake TR, Gorlova O, Rueda B, Martin JE, Alizadeh BZ, et al. (2010) Genome-wide association study of systemic sclerosis identifies CD247 as a new susceptibility locus. Nat Genet 42: 426-429.

61. Carmona FD, Martin JE, Beretta L, Simeon CP, Carreira PE, et al. (2013) The systemic lupus erythematosus IRF5 risk haplotype is associated with systemic sclerosis. PLoS One 8: e54419.

62. Martin JE, Broen JC, Carmona FD, Teruel M, Simeon CP, et al. (2012) Identification of CSK as a systemic sclerosis genetic risk factor through Genome Wide Association Study follow-up. Hum Mol Genet 21: 2825-2835.

63. Kristjansdottir G, Sandling JK, Bonetti A, Roos IM, Milani L, et al. (2008) Interferon regulatory factor 5 (IRF5) gene variants are associated with multiple sclerosis in three distinct populations. J Med Genet 45: 362-369.

64. Qiu W, Pham K, James I, Nolan D, Castley A, et al. (2013) The influence of non-HLA gene polymorphisms and interactions on disease risk in a Western Australian multiple sclerosis cohort. J Neuroimmunol 261: 92-97.

65. Gathungu G, Zhang CK, Zhang W, Cho JH (2012) A two-marker haplotype in the IRF5 gene is associated with inflammatory bowel disease in a North American cohort. Genes Immun 13: 351-355.

66. Dideberg V, Kristjansdottir G, Milani L, Libioulle C, Sigurdsson S, et al. (2007) An insertion-deletion polymorphism in the interferon regulatory Factor 5 (IRF5) gene confers risk of inflammatory bowel diseases. Hum Mol Genet 16: 3008-3016.

67. Li P, Lv H, Yang H, Qian JM (2013) IRF5, but not TLR4, DEFB1, or VDR, is associated with the risk of ulcerative colitis in a Han Chinese population. Scand J Gastroenterol 48: 1145-1151.

68. Sigurdsson S, Goring HH, Kristjansdottir G, Milani L, Nordmark G, et al. (2008) Comprehensive evaluation of the genetic variants of interferon regulatory factor 5 (IRF5) reveals a novel 5 bp length polymorphism as strong risk factor for systemic lupus erythematosus. Hum Mol Genet 17: 872-881.

69. Sigurdsson S, Nordmark G, Goring HH, Lindroos K, Wiman AC, et al. (2005) Polymorphisms in the tyrosine kinase 2 and interferon regulatory factor 5 genes are associated with systemic lupus erythematosus. Am J Hum Genet 76: 528-537.

70. Shin HD, Sung YK, Choi CB, Lee SO, Lee HW, et al. (2007) Replication of the genetic effects of IFN regulatory factor 5 (IRF5) on systemic lupus erythematosus in a Korean population. Arthritis Res Ther 9: R32.

71. Ferreiro-Neira I, Calaza M, Alonso-Perez E, Marchini M, Scorza R, et al. (2007) Opposed independent effects and epistasis in the complex association of IRF5 to SLE. Genes Immun 8: 429-438.

72. Hu W, Ren H (2011) A meta-analysis of the association of IRF5 polymorphism with systemic lupus erythematosus. Int J Immunogenet 38: 411-417.

73. Han SW, Lee WK, Kwon KT, Lee BK, Nam EJ, et al. (2009) Association of polymorphisms in interferon regulatory factor 5 gene with rheumatoid arthritis: a metaanalysis. J Rheumatol 36: 693-697.

74. Shimane K, Kochi Y, Yamada R, Okada Y, Suzuki A, et al. (2009) A single nucleotide polymorphism in the IRF5 promoter region is associated with susceptibility to rheumatoid arthritis in the Japanese population. Ann Rheum Dis 68: 377-383.

75. Lee YH, Bae SC, Choi SJ, Ji JD, Song GG (2013) Associations between interferon regulatory factor 5 polymorphisms and rheumatoid arthritis: a meta-analysis. Mol Biol Rep 40: 1791-1799.

76. Nordang GB, Viken MK, Amundsen SS, Sanchez ES, Flato B, et al. (2012) Interferon regulatory factor 5 gene polymorphism confers risk to several rheumatic diseases and correlates with expression of alternative thymic transcripts. Rheumatology (Oxford) 51: 619-626.

77. Kim YJ, Park JH, Kim I, Kim JO, Bae JS, et al. (2008) Putative role of functional interferon regulatory factor 5 (IRF5) polymorphism in rheumatoid arthritis in a Korean population. J Rheumatol 35: 2106-2118.

78. Maalej A, Hamad MB, Rebai A, Teixeira VH, Bahloul Z, et al. (2008) Association of IRF5 gene polymorphisms with rheumatoid arthritis in a Tunisian population. Scand J Rheumatol 37: 414-418.

79. Dieguez-Gonzalez R, Calaza M, Perez-Pampin E, de la Serna AR, Fernandez-Gutierrez B, et al. (2008) Association of interferon regulatory factor 5 haplotypes, similar to that found in systemic lupus erythematosus, in a large subgroup of patients with rheumatoid arthritis. Arthritis Rheum 58: 1264-1274.

80. Shimane K, Kochi Y, Yamada R, Okada Y, Suzuki A, et al. (2009) A single nucleotide polymorphism in the IRF5 promoter region is associated with susceptibility to rheumatoid arthritis in the Japanese population. Ann Rheum Dis 68: 377-383.

81. Sigurdsson S, Padyukov L, Kurreeman FA, Liljedahl U, Wiman AC, et al. (2007) Association of a haplotype in the promoter region of the interferon regulatory factor 5 gene with rheumatoid arthritis. Arthritis Rheum 56: 2202-2210.

82. Rueda B, Reddy MV, Gonzalez-Gay MA, Balsa A, Pascual-Salcedo D, et al. (2006) Analysis of IRF5 gene functional polymorphisms in rheumatoid arthritis. Arthritis Rheum 54: 3815-3819.

83. Yanagimachi M, Naruto T, Miyamae T, Hara T, Kikuchi M, et al. (2011) Association of IRF5 polymorphisms with susceptibility to macrophage activation syndrome in patients with juvenile idiopathic arthritis. J Rheumatol 38: 769-774.
